# New Insights into the Mechanism by Which the Pituitary Gland Copes with Hypoxia Stress Based on a Transcriptomic Analysis of *Megalobrama amblycephala*

**DOI:** 10.3390/genes15080987

**Published:** 2024-07-26

**Authors:** Ruilin Xie, Huandi Guo, Yuanyuan Luo, Wen Huang, Zhuohao Ruan, Wensheng Liu

**Affiliations:** 1 College of Marine Sciences, South China Agricultural University, Guangzhou 510642, China; m15876536141@163.com (R.X.); 18218260461@163.com (H.G.); 17334610172@163.com (Y.L.); 2 Laboratory of Aquatic Sciences, Key Laboratory of Animal Nutrition and Feed Science in South China of Ministry of Agriculture and Rural Affairs, Guangdong Key Laboratory of Animal Breeding and Nutrition, Institute of Animal Science, Guangdong Academy of Agricultural Sciences, Guangzhou 510640, China; huangwen549@126.com; 3 Guangdong Province Engineering Research Centre of Aquatic Immunization and Aquaculture Health Techniques, South China Agricultural University, Guangzhou 510642, China; 4 University Joint Laboratory of Guangdong Province, Hong Kong and Macao Region on Marine Bioresource Conservation and Exploitation, Guangzhou 510642, China

**Keywords:** *Megalobrama amblycephala*, transcriptomic, hypoxia, pituitary

## Abstract

Hypoxia is a common environmental stressor in aquatic ecosystems, and during the cultivation process, *Megalobrama amblycephala* is prone to death because it is hypoxia-intolerant, which brings huge economic losses to farmers. The pituitary gland is a crucial endocrine gland in fish, and it is mainly involved in the secretion, storage, and regulation of hormones. In the present study, we compared the transcriptional responses to serious hypoxia in the pituitary gland among hypoxia-sensitive (HS) and hypoxia-tolerant (HT) *M. amblycephala* and a control group that received a normal oxygen supply (C0). The fish were categorized according to the time required to lose balance during a hypoxia treatment. A total of 129,251,170 raw reads were obtained. After raw sequence filtering, 43,461,745, 42,609,567, and 42,730,282 clean reads were obtained for the HS, HT, and C0 groups, respectively. A transcriptomic comparison revealed 1234 genes that were differentially expressed in C0 vs. HS, while 1646 differentially expressed genes were obtained for C0 vs. HT. In addition, the results for HS vs. HT showed that 367 upregulated and 41 downregulated differentially expressed genes were obtained for a total of 408 differentially expressed genes. A KEGG analysis of C0 vs. HS, C0 vs. HT, and HS vs. HT identified 315, 322, and 219 enriched pathways, respectively. Similar hypoxia-induced transcription patterns suggested that the downregulated DEGs and enriched pathways were related to pathways of neurodegeneration in multiple diseases, pathways in cancer, thermogenesis, microRNAs in cancer, diabetic cardiomyopathy, and renin secretion. However, in the upregulated DEGs, the PI3K-Akt signaling pathway (C0 vs. HS), microRNAs in cancer (C0 vs. HT), and HIF-1 signaling pathway (HS vs. HT) were significantly enriched. There is a lack of clarity regarding the role of the pituitary gland in hypoxic stress. These results not only provide new insights into the mechanism by which pituitary tissue copes with hypoxia stress in *M. amblycephala* but also offer a basis for breeding *M. amblycephala* with hypoxia-resistant traits.

## 1. Introduction

Oxygen is indispensable for most organisms but some environmental changes can reduce its content [1]. Hypoxia is a common environmental stressor in aquatic ecosystems that has been proven to affect the behavior, growth, and physiological status of fish and can even lead to death. In recent years, instances of low oxygen in water bodies have become increasingly apparent due to various physical factors such as eutrophication and global warming. Therefore, hypoxia has become an increasingly important problem, leading to a great loss of aquaculture and a decrease in biodiversity [2,3]. Thus, studies about the regulation mechanism of hypoxia in fish are attracting more and more attention.

*M. amblycephala* belongs to Cypriniformes, Cyprinidate, and *Megalobrama*. It was the first fish species named by Chinese scientists after the founding of the People’s Republic of China and was also the first fish species successfully domesticated by Chinese aquatic scientists [4]. *M. amblycephala* has high acceptability in the consumer market and a high nutritional value. Due to its advantages of wide feeding habits, fast growth, a high survival rate, easy fishing, and moderate specifications, it has been vigorously promoted as an excellent herbivorous fish species, which, to a certain extent, has replaced and reduced the proportion of grass carp, adjusted the fishery structure, and reduced the cost and risk of breeding [5,6]. The total aquaculture yield of bream in China was 767,000 tons (with more than 90% of bream being *M. amblycephala*) in 2022 [7]. Compared with other cyprinid species such as grass carp, common carp, and crucian carp [8,9,10], *M. amblycephala* is a hypoxia-sensitive species, and hypoxia (below 0.5 mg/L), even for a short period, can be fatal [11,12]. Consequently, it is indispensable to determine the molecular mechanisms of hypoxia in fish.

So far, some genes related to hypoxia have been identified. Hypoxia-inducible factors (HIFs) are the key proteins in the process of the biological hypoxia response as they play an important role in its physiological regulatory network. Among them, *hif-1* is currently the transcription factor that people pay the most attention to. In most mammalian cells, *hif-1* is considered to be the primary regulator of transcription in response to hypoxia [13]. At present, there are over 100 confirmed genes downstream of *hif-1*. They are involved in red blood cell generation, iron metabolism, glucose metabolism, cell proliferation, angiogenesis, cell apoptosis, and other oxygen transport or metabolic adaptations related to hypoxia [14,15,16]. Currently, the hypoxia-related genes observed in fish mainly include hypoxia-inducible factor-1 (*hif-1*), vascular endothelial growth factor (*vegf*), transferrin (*tf*), erythropoietin (*epo*), IGF-binding protein-1 (*igfbp-1*), and nuclear factor kB (*nf-kB*) [17], and related research mainly focuses on molecular structures, cloning, and expression characteristics [18,19]. In a study of hypoxia in *M. amblycephala*, Shen found the molecular structures, expression characteristics, and regulatory mechanisms of three hypoxia-inducible factors, *hif-1α*, *hif-2α*, and *hif-3α*. It was discovered that they play different roles in the growth, development, and hypoxia response of *M. amblycephala* [20]. Chen cloned the *ho-2a* and *ho-2b* genes of heme oxygenase (HO) in *M. amblycephala* via the rapid amplification of cDNA ends and conducted a sequence analysis, spatiotemporal expression analysis, homology and molecular evolution analysis, whole-embryo in situ hybridization analysis, and hypoxia stress expression analysis [21]. In addition, research has found three oxygen-sensing genes in *M. amblycephala* (prolyl hydroxylase domain-containing protein 2 (*phd2*), prolyl hydroxylase domain-containing protein 3 (*phd3*), and hypoxia-inducible factor 1-alpha inhibitor (*hif1an*)), confirming the selective splicing of *phd3*, which is regulated by *hif-1α*. Thus, three SNP loci located in the *hif1an* gene were identified to be associated with the hypoxic trait in *M. amblycephala* [22,23]. However, the mechanism by which pituitary tissue copes with hypoxia stress in *M. amblycephala* is unclear.

Therefore, in order to understand the molecular mechanism of the hypoxia response at the transcriptional level in the *M. amblycephala* pituitary gland, hypoxia-sensitive (HS) and hypoxia-tolerant (HT) fish were subjected to hypoxia. Transcriptional differences in the pituitary gland were compared among HS, HT, and control (C0) fish based on RNA-seq in this study.

## 2. Materials and Methods

### 2.1. Ethics Statement

The Animal Care Committee of South China Agriculture University (Guangzhou, China) approved the current study under the trial registration number G026, and this research was carried out according to the Experimental Animal Management Law of China.

### 2.2. Animals and Feeding

**Figure 1:**
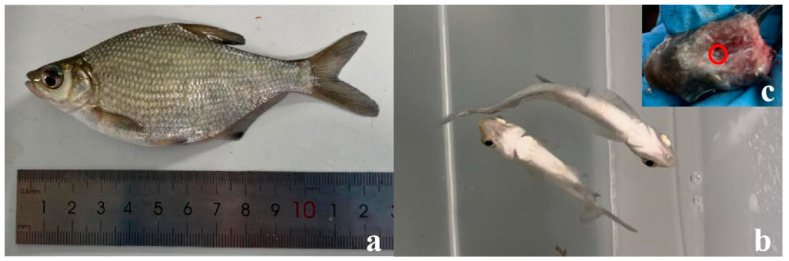
Pictures collected during the experiment. (**a**) *M. amblycephala* (9–11 cm and 18–20 g). (**b**) The symptoms of hypoxia in *M. amblycephala*. (**c**) A pituitary gland obtained from *M. amblycephala*.

Three hundred mixed-gender juvenile *M. amblycephala* (6–8 cm) from a full-sib family were purchased from the Shanghai Ocean University Department of Agriculture’s Center for Genetics and Breeding of *M. amblycephala*. All experimental fish were stocked in tanks (r = 1.5 m and h = 0.8 m) with water recirculation systems in the circulating water aquaculture base of the College of Marine Sciences, South China Agricultural University, and allowed to acclimatize for 30 days. The fish were fed about 3% of their total body weight. After one month of breeding, the *M. amblycephala* had grown to 9–11 cm (Figure 1a). The water quality indicators during this period are shown in Table 1.

### 2.3. Hypoxia Challenge and Sample Preparation

**Figure 2:**
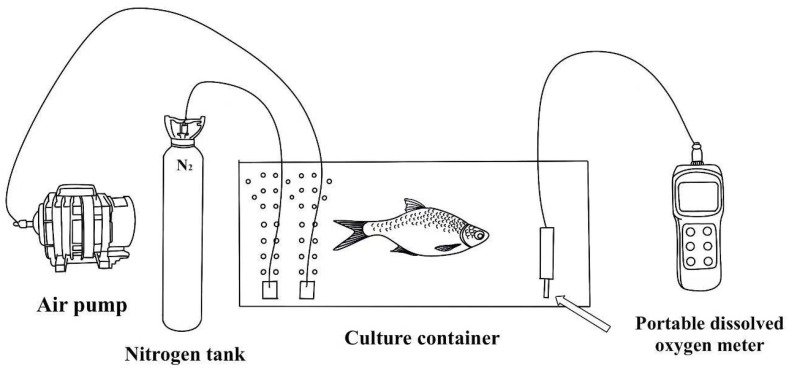
The hypoxia device used in this experiment.

After one month of temporary care, healthy *M. amblycephala* with the same specifications were randomly divided into 6 groups (experimental and control groups, each with 3 parallel groups). Each group contained 30 healthy and undamaged individuals. The dissolved oxygen (DO) was continuously monitored using an AR8406 portable dissolved oxygen analyzer (Smart Sensor Ltd., Hongkong) (Figure 2). In the present study, the loss of equilibrium (LOE) was chosen as the indicator of hypoxia in consideration of its physiological and behavioral factors (Figure 1b).

The control group received a normal oxygen supply, ensuring that the DO was 7 ± 0.5 mg/L (22.5 °C). For the experimental group, nitrogen gas was introduced to reduce the dissolved oxygen in the water, and the DO was maintained at 0.3 ± 0.1 mg/L (23 °C) during the hypoxia stress stage. After 60 min of hypoxia, some fish started to lose balance. The first ten fish that lost their balance were removed and immediately sampled as hypoxia-sensitive (HS). As the hypoxia time was extended, about half of the fish lost their balance and were continuously removed, and the last ten fish were regarded as hypoxia-tolerant (HT). The same procedure was performed on all three parallel groups. Subsequently, 10 fish were randomly selected from the three control groups as the normoxic control group (C0).

After measuring the growth data of the selected individuals, their pituitary glands were removed, immediately stored in liquid nitrogen, and transferred to a refrigerator at −80 °C for preservation (Figure 1c). Nine tubes were used for RNA isolation, each containing at least two fish pituitary glands.

### 2.4. RNA Extraction, Library Construction, and Sequencing

Total RNA was extracted using a TRIzol reagent kit (Invitrogen, Carlsbad, CA, USA) according to the manufacturer’s protocol. RNA quality was assessed using an Agilent 2100 Bioanalyzer (Agilent Technologies, Palo Alto, CA, USA) and checked using RNase-free agarose gel electrophoresis. After total RNA was extracted, eukaryotic mRNA was enriched using Oligo (dT) beads. Then, the enriched mRNA was fragmented into short fragments using a fragmentation buffer and reverse-transcribed into cDNA using an NEBNext Ultra RNA Library Prep Kit for Illumina (NEB #7530, New England Biolabs, Ipswich, MA, USA). The purified double-stranded cDNA fragments were end-repaired, a base was added, and the fragments were ligated to Illumina sequencing adapters. The ligation reaction was purified with AMPure XP Beads (1.0×) and amplified via a polymerase chain reaction (PCR). The resulting cDNA library was sequenced using Illumina Novaseq6000 from Gene Denovo Biotechnology Co. (Guangzhou, China).

### 2.5. Differentially Expressed Genes (DEGs)

The differential RNA expression between two groups was analyzed using DESeq2 [24] software, and the differential expression between two samples was analyzed using edgeR [25]. The genes/transcripts with a false discovery rate (FDR) below 0.05 and |log_2_FC| ≥ 2 were considered to be differentially expressed.

### 2.6. GO and KEGG Enrichment Analyses

Gene Ontology (GO) [26] is an international standardized gene functional classification system that offers a dynamically updated controlled vocabulary and a strictly defined concept to comprehensively describe the properties of genes and their products in any organism. GO has the following three ontologies: molecular functions, cellular components, and biological processes. The basic unit of GO is a GO term. Each GO term belongs to a type of ontology. A GO enrichment analysis provides all GO terms that are significantly enriched in DEGs compared with the genome background, and filters the DEGs that correspond with biological functions. First, all DEGs were mapped to GO terms in the Gene Ontology database (http://www.geneontology.org/, accessed on 30 January, 2024), and gene numbers were calculated for every term. GO terms in DEGs that were significantly enriched compared with the genome background were defined using a hypergeometric test. The calculated *p*-values were subjected to the FDR correction, taking an FDR ≤ 0.05 as a threshold. GO terms meeting this condition were defined as significantly enriched GO terms in DEGs. This analysis was able to recognize the main biological functions of the DEGs.

Genes usually interact with each other to play roles in certain biological functions. A pathway-based analysis helps to further understand genes’ biological functions. KEGG [27] is the major public pathway-related database. The pathway enrichment analysis identified significantly enriched metabolic or signal transduction pathways in the DEGs compared with the genome background. The calculated *p*-values were subjected to the FDR correction, taking an FDR ≤ 0.05 as a threshold. Pathways meeting this condition were defined as significantly enriched pathways in DEGs.

### 2.7. Real-Time PCR

To validate the RNA-seq results, real-time PCR (RT-PCR) was carried out with SYBR Green I Master Mix (Roche). Six randomly selected DEGs (calpain-1 (*capn1*), heme oxygenase 1 (*hmox1*), hypoxia-inducible factor 1 subunit alpha inhibitor (*hif1an*), insulin receptor substrate 2 (*Irs2*), fascin 1 (*fscn1*), and insulin-like growth factor 1 (*igf1*)) were used to determine the expression patterns. ACTB (*β-actin*) was used as the internal gene, as previously described [28]. RT-PCR was performed using a total volume of 20 μL and a QuantStudio™ 6 Flex Real-Time PCR System (ABI, NewYork, NY, USA) according to the manufacturer’s instructions. The reaction conditions were 95 °C for 30 s, followed by 40 cycles of 95 °C for 10 s, 60 °C for 30 s, 95 °C for 15 s, 60 °C for 60 s, and 95 °C for 15 s. The sequences of the primers used in this study are listed in Table 2. The relative expression was calculated using the comparative CT (ΔΔCT) method. Statistical significance (*p* < 0.05) was determined using a one-way ANOVA and Duncan’s multiple range tests using SPSS 17.0.

## 3. Results

### 3.1. Phenotype Analysis of M. amblycephala during the Hypoxia Challenge

Before the beginning of the official experiment, a low-oxygen tolerance test was conducted on farmed *M. amblycephala* where the dissolved oxygen was reduced by introducing nitrogen gas. About half an hour later, when the DO decreased to 0.3 mg/L, the first *M. amblycephala* started gasping for air at the surface of the water. The DO was reduced from 0.3 mg/L to 0.2 mg/L. This lasted for about half a minute, during which more than one-third of the *M. amblycephala* rose to the surface and swallowed the air, exhibiting behaviors such as wild swimming, restlessness, and even jumping out of the water. When the DO reached 0.2 mg/L, more than half of the *M. amblycephala* almost simultaneously lost their balance, so it was confirmed that this point was the dissolved oxygen threshold where the *M. amblycephala* lost their equilibrium (0.2 mg/L). During the official experiment, a DO level of 0.3 ± 0.1 mg/L was maintained for hypoxia stress. According to the method used by Chen [29], the first 10 fish in all three experimental groups that lost their balance were added to the hypoxia-sensitive group (HS), while the last 10 fish were added to the hypoxia-tolerant group (HT). Subsequently, 10 fish were randomly selected from each of the three control groups as the normal oxygen control group (C0), and the growth data of the selected individuals were measured (Table 3).

### 3.2. Acquisition of Differentially Expressed Genes in the Pituitary in Response to Hypoxia

The pituitary glands were subjected to high-throughput sequencing using the Illumina Hiseq2000 platform, and the final sequencing yield statistics of the nine cDNA libraries are shown in Table 4. The results showed that a total of 129,251,170 raw reads were obtained. After removing and trimming the low-quality reads, adaptors, poly-A tails, and reads containing > 5% unknown nucleotides, 43,461,745, 42,609,567, and 42,730,282 clean reads were obtained for HS (the hypoxia-sensitive group), HT (the hypoxia-tolerant group), and C0 (the normal oxygen control group), respectively. All Q20 values were greater than 98.41% and the GC proportion was greater than 43.82%, which indicated good sequencing quality and that these data could be used for the subsequent analysis. 

**Figure 3:**
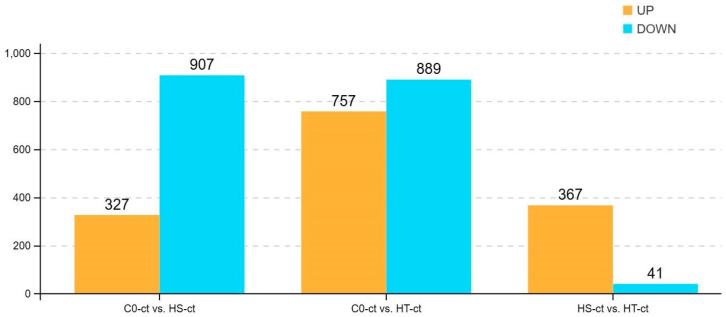
The numbers of differentially expressed genes in the three groups of *M. amblycephala*. The *x*-axis indicates the groups of *M. amblycephala*, and the *y*-axis indicates the numbers of differentially expressed genes.

The differentially expressed genes in the three groups were screened according to the conditions of a *p*-value < 0.05 and |log_2_FC| > log_2_(2). The obtained results showed that a total of 1234 differentially expressed genes were obtained for C0 vs. HS (including 327 upregulated genes and 907 downregulated genes), while 1646 differentially expressed genes were obtained for C0 vs. HT (757 upregulated genes and 889 downregulated genes). In addition, the results for HS vs. HT were different, as 367 upregulated and 41 downregulated differentially expressed genes were obtained for a total of 408 differentially expressed genes (Figure 3).

Note: The total raw reads, total clean reads, Q20 ratio, and GC ratio are all averages. The Q20 proportion refers to the proportion of bases with masses of no fewer than 20 after filtering. The GC ratio indicates the proportion of filtered G and C bases in the total number of bases.

### 3.3. GO Functional Classification of Differentially Expressed Genes

**Figure 4:**
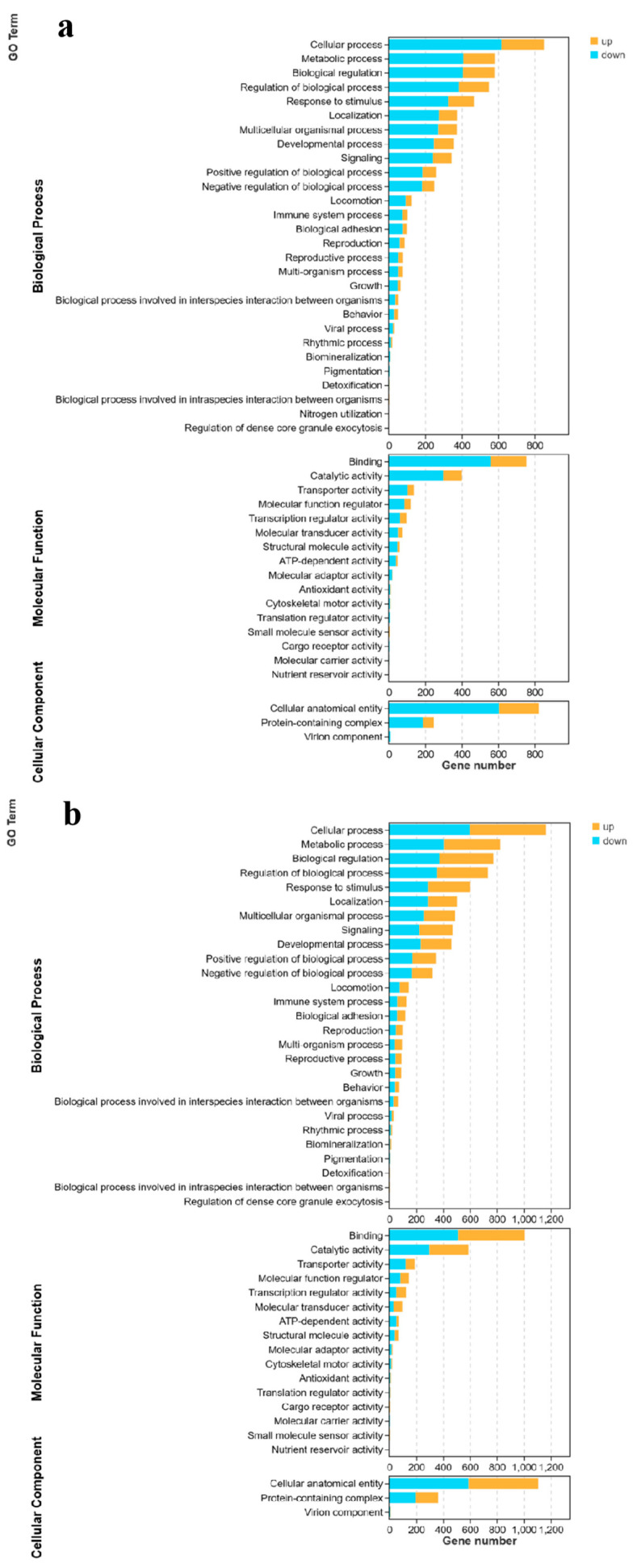

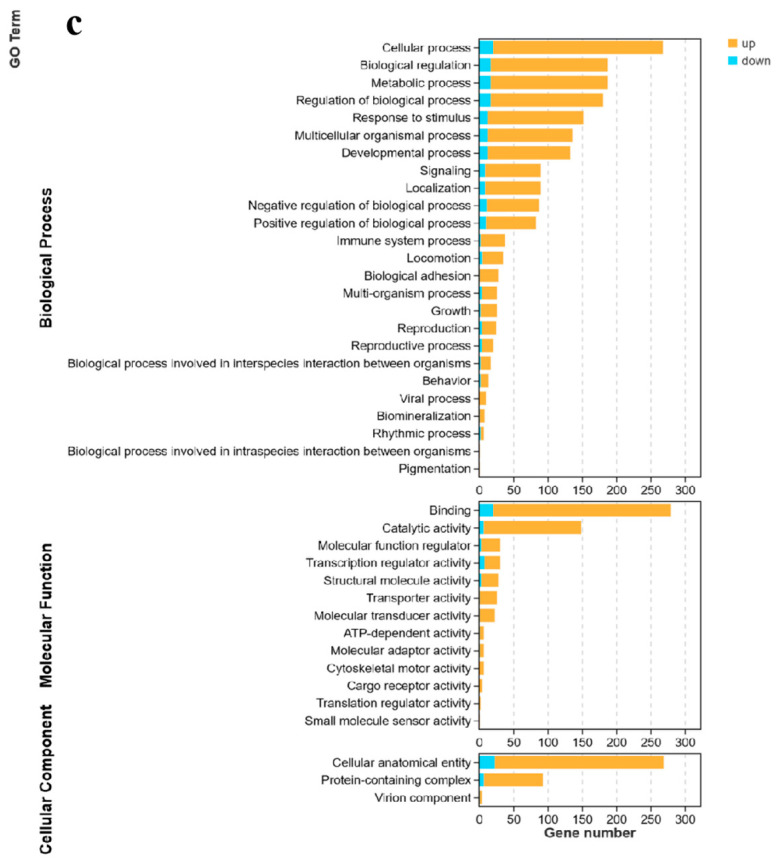
GO classification of the differentially expressed genes in the three groups of *M. amblycephala*. (**a**) C0 vs. HS. (**b**) C0 vs. HT. (**c**) HS vs. HT.

GO is a database suitable for various species. It defines and describes gene functions while constantly updating their vocabulary. GO functional annotations are mainly divided into the following three functional categories: biological processes, cellular components, and molecular functions. The results of the DEG annotations for the three groups of *M. amblycephala* are shown in Figure 4. Most of the DEGs were enriched in biological processes and had relatively few annotations related to cellular components.

In the three groups of *M. amblycephala*, the DEGs involved in the biological process were mainly involved in the following five GO pathways: cellular processes, metabolic processes, biological regulation, regulation of biological processes, and responses to stimuli. The main GO molecular function categories were binding, catalytic activity, transporter activity, molecular function regulators, and transcription regulator activity. In addition, the proportions of cellular anatomical entities and protein-containing complexes were high among the cellular components.

### 3.4. Differentially Expressed Genes Related to Hypoxia Stress Obtained from KEGG Analysis

Within living organisms, various genes interact to carry out biological functions. Leveraging KEGG facilitated a deeper exploration of the intricate behaviors of genes at the biological level. Using KEGG annotation information, sequences could be further annotated, and the pathway-based analysis aided in a better comprehension of the biological functions of the genes. After correcting for multiple tests, pathways with *q-values* < 0.05 were considered to be significantly different among the differentially expressed genes.

**Figure 5:**
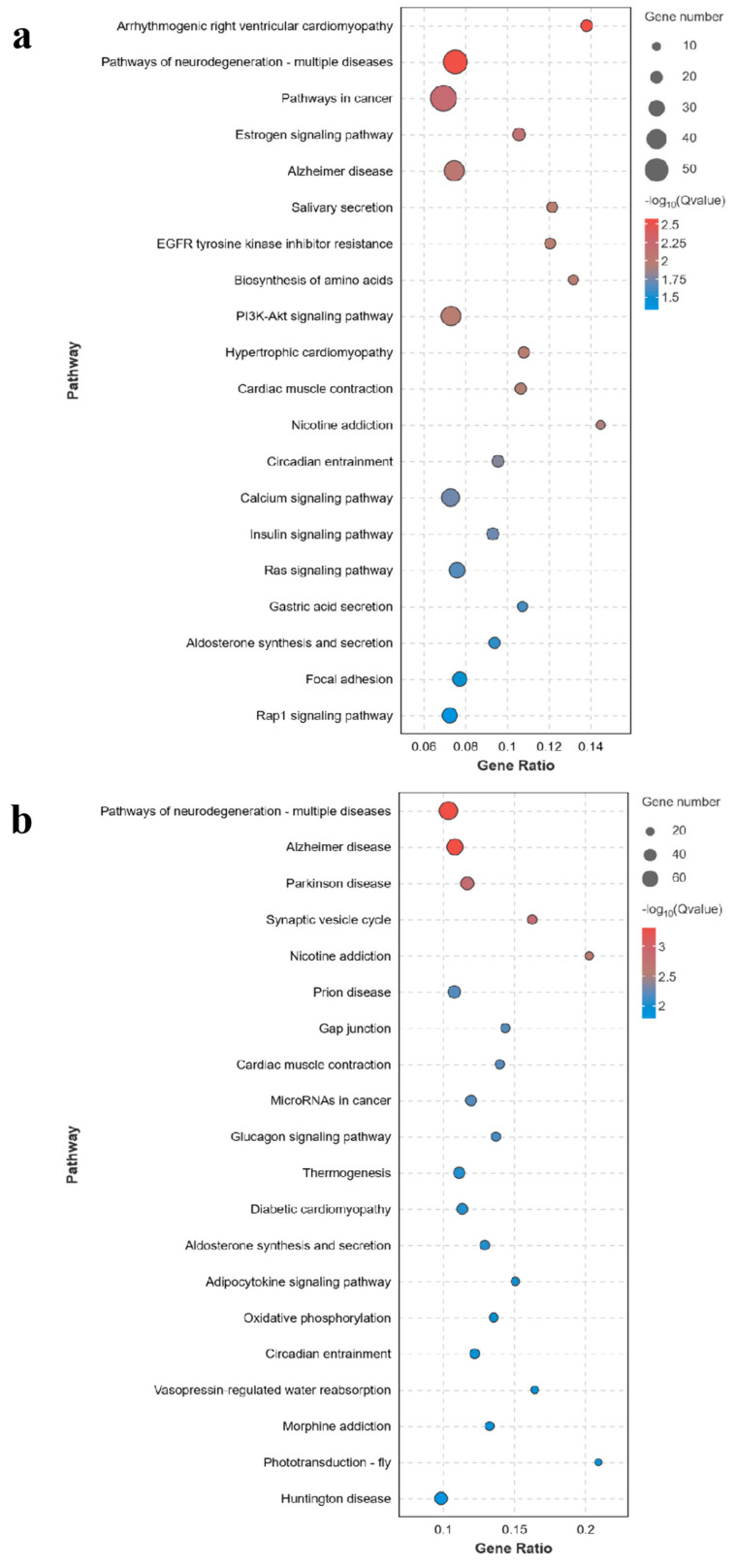

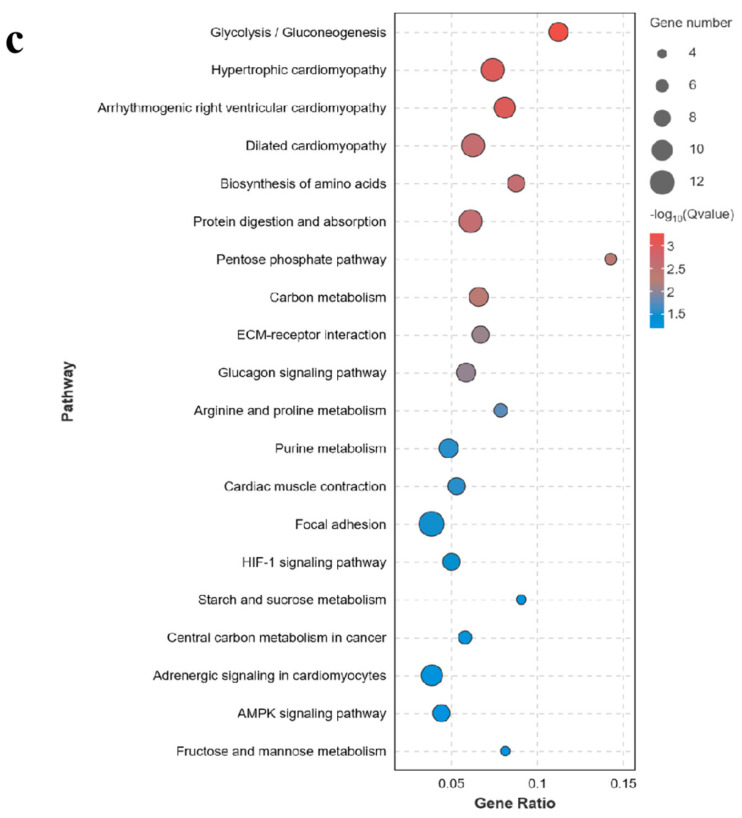
KEGG enrichment of the differentially expressed genes in the three groups of *M. amblycephala*. (**a**) C0 vs. HS. (**b**) C0 vs. HT. (**c**) HS vs. HT.

The KEGG analysis of C0 vs. HS, C0 vs. HT, and HS vs. HT indicated 315, 322, and 219 enriched pathways, respectively, and the 20 most significant differences are shown in Figure 5a–c. The enriched pathways associated with hypoxia stress are provided in Table 5a–c. In the downregulated DEGs, the enriched pathways were related to pathways of neurodegeneration in multiple diseases, pathways in cancer, thermogenesis, microRNAs in cancer, diabetic cardiomyopathy, and renin secretion. In the upregulated DEGs, the PI3K-Akt signaling pathway (C0 vs. HS), microRNAs in cancer (C0 vs. HT), and HIF-1 signaling pathway (HS vs. HT) were significantly enriched (Table 6).

### 3.5. Validation of the RNA-seq Data for Differentially Expressed Genes Associated with Hypoxia Stress

**Figure 6:**
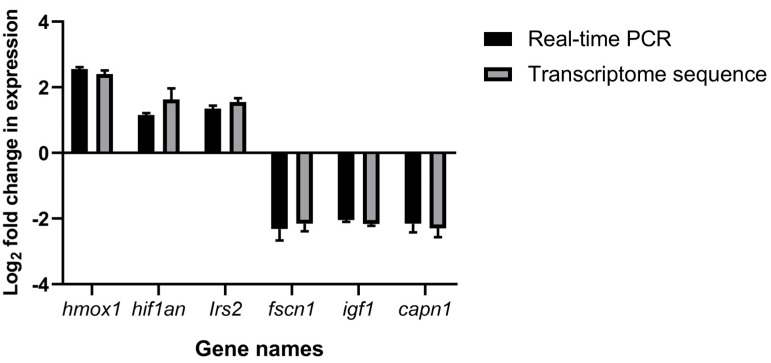
Validation of RNA-seq data using quantitative real-time polymerase chain reaction (RT-PCR).

In this study, the relative mRNA expression of five genes associated with hypoxia stress (*capn1*, *hmox1*, *hif1an*, *Irs2*, and *fscn1*) in *M. amblycephala* was tested using real-time PCR, and one differentially expressed gene (*igf1*) was also randomly selected for genetic validation. After a T-test analysis using GraphPad Prism 8.0 software, a bar chart was drawn according to the analysis results, and the results showed the standard error of the mean. The results indicated that the RT-PCR data were consistent with and validated the RNA-seq data (Figure 6).

## 4. Discussion

In recent years, the phenomenon of water hypoxia has become more and more obvious. *M. amblycephala* is not tolerant to low oxygen and is prone to hypoxia caused by dissolved oxygen changes in water during the process of breeding. Evaluation indicators of low-oxygen tolerance traits in fish have been reported by many researchers. The critical oxygen tension for the routine oxygen consumption rate (Pcrit) is the minimum oxygen level required to sustain routine oxygen consumption, which can reflect the ability of environments with changing dissolved oxygen levels to maintain the conventional metabolism of fish [30]. Aquatic surface respiration (ASR) is a mode of behavioral adaptation in fish that is observed following altered oxygen levels. It generally manifests as fish floating near the water surface to breathe in order to obtain more oxygen [31,32,33]. The oxygen threshold for the loss of equilibrium (LOE) represents when fish are unable to maintain balance due to oxygen stress. Fish can maintain a complete physiological energy supply above the dissolved oxygen level of the LOE. However, once the LOE is reached, many physiological metabolic pathways are activated to cope with high energy demands [34,35]. Pcrit, ASR, and the LOE are all considered to be important indicators when evaluating the hypoxia tolerance of fish. Considering the influence of physiological and behavioral factors and the feasibility of experimental manipulation, the LOE was more appropriate than the other two indicators. Therefore, the LOE was selected as the index to evaluate the hypoxia tolerance of the fish in this study. The LOE50 of *Megalobrama* is generally 0.3 mg/L [36]. Due to the fact that the *M. amblycephala* selected for this experiment belonged to the new variety “*M. amblycephala* of Pujiang No.2”, which was independently developed by Shanghai Ocean University, they had a certain degree of low-oxygen tolerance. During the experiment, the initial oxygen concentration that caused the farmed *M. amblycephala* to lose balance and flip over was 0.3 mg/L.

In this study, the pituitary glands of the *M. amblycephala* were removed and used for a transcriptomic analysis. In previous studies, the main tissues used for transcriptome research were the hepatopancreas [37,38,39,40], muscle [41,42], and gill [43]; the pituitary glands of fish have never been used in a transcriptomic analysis. The pituitary gland is a key tissue that regulates the neural axis of fish, playing an important role in the regulation of hormone secretion.

Based on the transcriptome analysis, we found that the numbers of downregulated DEGs were higher than those of upregulated DEGs in both the HS and HT groups, which were subjected to hypoxia. This agreed with previous studies based on transcriptomes [44,45]. It may be speculated that the predominant hypoxia response in the pituitary gland was mediated through gene repression rather than gene induction. The GO functional enrichment analysis showed that in the three groups of *M. amblycephala*, the DEGs were involved in similar GO pathways, but the expression trends of the groups were different as the DEGs in the SB group were mainly upregulated. The KEGG analysis revealed that some of the DEGs that were significantly enriched in some of the pathways may have been related to hypoxia. In the downregulated DEGs, the enriched pathways were related to pathways of neurodegeneration in multiple diseases [46], pathways in cancer, microRNAs in cancer [47], thermogenesis [48,49], diabetic cardiomyopathy [50], and renin secretion [51,52,53]. In the upregulated DEGs, the PI3K-Akt signaling pathway (C0 vs. HS) [54,55], microRNAs in cancer (C0 vs. HT), and HIF-1 signaling pathway (HS vs. HT) [56,57] were significantly enriched. It has been reported that some genes such as prolyl hydroxylase 2 (*phd2*), *hif-1a*, *hif-2a*, nitric oxide synthase 2 (*nos2*), and vascular endothelial growth factor (*vegf*) have evolutionarily undergone positive selection to adapt to high-altitude weather [28,58,59]. There are also many studies indicating that fascin 1 (*fscn1*), heme oxygenase 1 (*hmox1*), and enolase are associated with low-oxygen stress [60,61,62,63]. In this study, we found that the expression of *vegf-a*, *nos2*, *fscn1*, *hmox1*, *eno3,* and *hif1an* was significantly different in the three groups, indicating that these biological processes or genes may be related to hypoxia adaptation in *M. amblycephala*.

The different responses to hypoxia at the transcript level in the pituitary gland of *M. amblycephala* may have been a result of differences in hypoxia tolerance between individuals, which is the basis for the genetics and breeding of fish with hypoxia tolerance.

## 5. Conclusions

In this study, the low-oxygen tolerance performance of breeding *M. amblycephala* was tested and transcriptome sequencing was performed on pituitary glands from *M. amblycephala* under hypoxia stress and normoxia. The dissolved oxygen threshold where the *M. amblycephala* lost their equilibrium was found to be 0.2 mg/L. A transcriptomic comparison revealed 1234 genes that were differentially expressed in C0 vs. HS, while 1646 differentially expressed genes were obtained in C0 vs. HT. In addition, the results of HS vs. HT showed that a total of 408 differentially expressed genes were obtained. The KEGG analysis results of the three groups showed that similar hypoxia-induced transcription patterns were suggested by the downregulated DEGs. The enriched pathways were related to pathways of neurodegeneration in multiple diseases, pathways in cancer, thermogenesis, etc., but in the upregulated DEGs, the PI3K-Akt signaling pathway, microRNAs in cancer, and the HIF-1 signaling pathway were significantly enriched. The adaptation of *M. amblycephala* under hypoxia stress may be due to the expression levels of these differentially expressed genes. This study not only provides the first insights into the mechanism by which pituitary tissue copes with hypoxia stress in *M. amblycephala* but also offers a basis for breeding *M. amblycephala* with hypoxia-resistant traits.

## Figures and Tables

**Table 1:** The water quality indicators during the breeding period.

	Temperature (°C)	DO (mg/L)	pH	Ammonia Nitrogen (mg/L)	Nitrite (mg/L)
First Week	24.2 ± 0.4	7.38	9.0	0.2	0.005
Second Week	23.3 ± 0.2	8.15	9.2	0.2	0.005
Third Week	23.2 ± 0.4	8.61	8.5	0.2	0.010
Fourth Week	22.4 ± 0.1	6.89	8.6	0.2	0.010

**Table 2:** The sequences of the primers used in this study.

Gene Name	Primer Name	Sequence
*capn1*	*capn1*-F	CTTTGTTCCCAGCCAGTAGT
	*capn1*-R	GCAGGAGGTTATCGTTTAGAG
*hmox*	*hmox*-F	ATCCACGAAAAGCAAACAA
	*hmox*-R	AAGTAGACGGGCTGAACG
*hif1an*	*hif1an*-F	TTGTGGTGGATTTCCTTGG
	*hif1an*-R	GCTGGTGTTACATTGCCTTC
*Irs2*	*Irs2*-F	ATGAGAATGGCGAGTCCG
	*Irs2*-R	AGGCACGAGTCCAAATGTA
*fscn1*	*fscn1*-F	AAGAGCCATCTGGGGAGG
	*fscn1*-R	CTGGGCGAAGCAGGTAAT
*igf1*	*igf1*-F	TTTGCGGTACTTTGCTTGC
	*igf1*-R	CATTTGTCATTCCGTTTCTATC
*β-actin*	*β-actin*-F	CAGCAGATGTGGATTAGCAA
	*β-actin*-R	CAGTTTGAGTCGGCGTGA

**Table 3:** The growth data of the selected *M. amblycephala* in the three groups.

Sample		Average Body Length (cm)	Average Body Weight (g)
Hypoxia-sensitive group (HS)	HS1	10.1 ± 0.1	20.5 ± 0.6
	HS2	9.5 ± 0.6	17.6 ± 0.8
	HS3	10.7 ± 0.6	21.3 ± 0.5
Hypoxia-tolerant group (HT)	HT1	10.3 ± 0.4	20.8 ± 0.4
	HT2	9.6 ± 0.5	18.9 ± 0.2
	HT3	9.8 ± 0.2	21.3 ± 0.8
Normal oxygen control group (C0)	C1	9.2 ± 0.4	18.4 ± 0.8
	C2	9.3 ± 0.7	19.2 ± 0.2
	C3	9.5 ± 0.4	18.9 ± 0.5

**Table 4:** Sequencing yield statistics of the *M. amblycephala* in the three groups.

Sample		Total Raw Reads	Total Clean Reads	Q20 Ratio after Filtration (%)	GC Ratio (%)
Hypoxia-sensitive group (HS)	HS1-ct	41.60	41.44	98.81	45.76
	HS2-ct	42.70	42.58	98.89	47.06
	HS3-ct	46.57	46.37	98.49	47.20
	Average	43.62	43.46		
Hypoxia-tolerant group (HT)	HT1-ct	46.50	46.35	98.85	46.40
	HT2-ct	41.00	40.87	98.83	46.34
	HT3-ct	40.76	40.62	98.50	47.17
	Average	42.75	42.61		
Normal oxygen control group (C0)	C1-ct	48.48	48.33	98.80	44.39
	C2-ct	43.09	42.94	98.43	44.66
	C3-ct	37.05	36.93	98.41	43.82
	Average	42.88	42.73		
Total		129.25	128.8		

Note: The total raw reads, total clean reads, Q20 ratio, and GC ratio are all averages. The Q20 proportion refers to the proportion of bases with masses of no fewer than 20 after filtering. The GC ratio indicates the proportion of filtered G and C bases in the total number of bases.

**Table 5:** Hypoxia-related pathways in the KEGG analysis: (a) C0 vs. HS; (b) C0 vs. HT; (c) HS vs. HT.

Ko ID	KEGG Pathway	Gene Number	q-Value
**(a)**			
ko05022	Pathways of neurodegeneration—multiple diseases	50	0.002621
ko05200	Pathways in cancer	55	0.005909
ko05010	Alzheimer disease	40	0.008931
ko01521	EGFR tyrosine kinase inhibitor resistance	14	0.010725
ko04151	PI3K-Akt signaling pathway	38	0.010725
ko05410	Hypertrophic cardiomyopathy	16	0.010725
ko04910	Insulin signaling pathway	18	0.018797
ko04971	Gastric acid secretion	13	0.023564
ko04925	Aldosterone synthesis and secretion	16	0.027028
ko00561	Glycerolipid metabolism	9	0.045174
ko04714	Thermogenesis	22	0.045174
**(b)**			
ko05022	Pathways of neurodegeneration—multiple diseases	69	0.000473
ko05010	Alzheimer disease	58	0.000473
ko05206	MicroRNAs in cancer	29	0.006187
ko04714	Thermogenesis	32	0.008774
ko05415	Diabetic cardiomyopathy	30	0.008774
ko04925	Aldosterone synthesis and secretion	22	0.008774
ko04920	Adipocytokine signaling pathway	16	0.009259
ko00190	Oxidative phosphorylation	19	0.010375
ko04722	Neurotrophin signaling pathway	21	0.018898
ko04924	Renin secretion	16	0.028885
ko05208	Chemical carcinogenesis—reactive oxygen species	29	0.035122
ko04961	Endocrine and other factor-regulated calcium reabsorption	12	0.035802
ko04932	Non-alcoholic fatty liver disease	25	0.039190
ko04152	AMPK signaling pathway	20	0.041482
ko05214	Glioma	14	0.042298
ko05014	Amyotrophic lateral sclerosis	42	0.044880
**(c)**			
ko05410	Hypertrophic cardiomyopathy	11	0.000909
ko00030	Pentose phosphate pathway	5	0.004514
ko04066	HIF-1 signaling pathway	8	0.034028

**Table 6:** (a) Differentially expressed genes related to hypoxia stress in C0 vs. HS; (b) differentially expressed genes related to hypoxia stress in C0 vs. HT; (c) differentially expressed genes related to hypoxia stress in HS vs. HT.

**(a)**			
**Ko ID**	**Gene Abbreviation**	**Gene**	**Log2 Fold Change**
K01367	*CAPN1*	Calpain-1	−2.8205
K02183	*CALM1*	Calmodulin	−2.0736
K04961	*RYR1*	Ryanodine receptor 1	−2.8205
K05199	*GRIA3*	Glutamate receptor 3	−2.0555
K13241	*NOS2*	Nitric oxide synthase, inducible	−2.0357
K05448	*VEGFA*	Vascular endothelial growth factor A	−1.1658
K05459	*IGF1*	Insulin-like growth factor 1	−2.1498
K08765	*CPT1A*	Carnitine O-palmitoyltransferase 1, liver isoform	−1.9533
K04356	*NTF3*	Neurotrophin 3	1.9761
**(b)**			
**Ko ID**	**Gene Abbreviation**	**Gene**	**Log2 Fold Change**
K23551	*FSCN1*	Fascin 1	−3.8669
K05852	*PLN*	Phospholamban	−4.6055
K05262	*ADCYAP*	Pituitary adenylate cyclase-activating polypeptide	−4.8438
K00510	*hmox1*	Heme oxygenase 1	3.9614
K04361	*EGFR*	Epidermal growth factor receptor	1.1630
K07187	*IRS2*	Insulin receptor substrate 2	1.4813
**(c)**			
**Ko ID**	**Gene Abbreviation**	**Gene**	**Up/Down**
K01689	*ENO3*	Enolase	2.2894
-	*hif1an*	HIF-1 alpha subunit inhibitor	1.3558
K00134	*GAPDH*	Glyceraldehyde 3-phosphate dehydrogenase	3.0927

## Data Availability

The original contributions presented in the study are included in the article, further inquiries can be directed to the corresponding authors.
